# Potential role of electroencephalographic monitoring for diagnosis and treatment of local anesthetic systemic toxicity during general anesthesia: a case report

**DOI:** 10.1186/s40981-024-00763-8

**Published:** 2024-12-28

**Authors:** Ryo Wakabayashi, Seiichi Azuma, Saori Hayashi, Yuji Ueda, Masaki Iwakiri, Masaaki Asamoto, Kanji Uchida

**Affiliations:** Department of Anesthesiology and Pain Relief Center, The University of Tokyo Hospital, 7-3-1 Hongo, Bunkyo-ku, Tokyo 113-8655 Japan

**Keywords:** Electroencephalogram, Electrographic seizures, Epileptiform discharges, Local anesthetic systemic toxicity

## Abstract

**Background:**

Local anesthetic systemic toxicity (LAST) is a rare but potentially life-threatening complication. Under general anesthesia, neurological signs are often masked, delaying diagnosis and increasing the risk of sudden cardiovascular collapse. Therefore, early detection methods are critically needed.

**Case presentation:**

A 48-year-old male patient (height: 182 cm, weight: 98 kg) underwent resection of a mediastinal goiter. He received 10 mL of 4% lidocaine for topical airway anesthesia and 20 mL of 1% lidocaine with 1:100,000 epinephrine for chest wall anesthesia. Thirty minutes after airway anesthesia, continuous theta waves appeared on the frontal electroencephalogram (EEG), which were enhanced following chest wall anesthesia. These waves transitioned into a repeating pattern and evolved into sharp periodic discharges. After administering 150 mL of 20% lipid emulsion, the EEG normalized.

**Conclusions:**

This case highlights that EEG monitoring during general anesthesia may facilitate the early detection of LAST and provide real-time feedback on treatment efficacy.

## Background

Local anesthetic systemic toxicity (LAST) is a rare but potentially life-threatening complication associated with the use of local anesthetics [1]. Clinical manifestations of LAST encompass a spectrum of neurological and cardiovascular symptoms, starting with mild symptoms (e.g., dizziness, tinnitus, and conduction disturbances) and progressing to severe symptoms (e.g., seizures, coma, and cardiac arrest) [2]. Early recognition of LAST and prompt treatment with lipid emulsion, which promotes the redistribution of local anesthetics from the brain and heart to peripheral tissues such as muscle and liver, are critical for ensuring patient safety [1].

In approximately 70% of cases, neurological symptoms precede the onset of cardiovascular symptoms [2]. However, under general anesthesia, particularly when neuromuscular blockade is used, the typical neurological signs of toxicity are often masked. This delay in recognizing LAST increases the risk of sudden cardiovascular collapse [3]. These challenges underscore the need for clinical advancements to enable the early detection of LAST during general anesthesia.

Here, we report a case in which electroencephalogram (EEG) monitoring under general anesthesia appeared to facilitate the early detection of LAST and provide real-time feedback on treatment efficacy. Written informed consent for publication of this case report was obtained from the patient.

## Case presentation

A previously healthy 48-year-old male patient (height: 182 cm, weight: 98 kg) was scheduled to undergo resection of a mediastinal goiter. Preoperative laboratory investigations were within normal limits. A chest radiograph revealed no lung infiltrates or cardiomegaly. An electrocardiogram (ECG) showed normal sinus rhythm at 60 bpm. Computed tomography of the neck and chest revealed a tumor measuring 191 × 84 × 68 mm, extending from the right thyroid lobe to the anterior region of the tracheal bifurcation. The tumor caused leftward deviation and compression of the trachea in the cervical region and posterior compression in the thoracic region. The patient presented with dyspnea in the supine position, although arterial oxygen saturation was maintained at 99% on room air. No central nervous system (CNS) abnormalities were noted preoperatively.

The patient received no premedication. In the operating room, standard biometric monitoring was performed. Additionally, invasive arterial blood pressure monitoring, processed EEG, and acceleromyography at the adductor pollicis muscle were implemented. Processed EEG monitoring was conducted using a BIS Quatro sensor (Medtronic, Minneapolis, MN) placed on the left forehead, with electrode impedance maintained at 5 kΩ or less throughout the procedure. EEG waveform data were recorded in a database at a sampling rate of 250 Hz and analyzed offline using MATLAB R2024a (MathWorks, Natick, MA). The power spectrum and spectrogram were generated using the Chronux *mtspecgramc* function with the following parameters: a window length of 3 s, a 0.5-s overlap, a time-bandwidth product of 3, and 5 tapers.

**Fig. 1:**
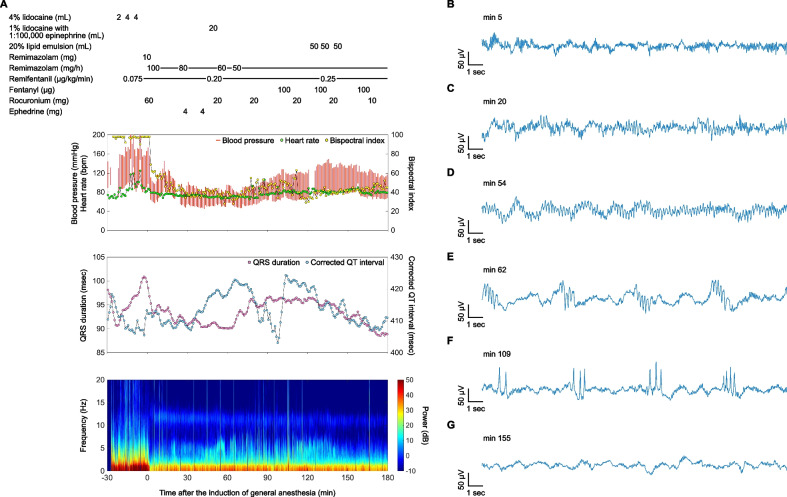
Anesthetic record with a spectrogram of the frontal electroencephalogram (EEG), and time-domain frontal EEG signatures. **A** Anesthetic record and spectrogram throughout the course of events, with time 0 marking the induction of general anesthesia. EEG waveform (**B**) after induction of general anesthesia with remimazolam, showing delta and alpha oscillations; **C** 30 min after topical airway anesthesia with a total of 10 mL of 4% lidocaine, displaying the emergence of continuous theta waves; **D** immediately after local anesthesia of the chest wall with 20 mL of 1% lidocaine containing 1:100,000 epinephrine, illustrating enhanced continuous theta waves; **E** after the transition of theta waves to a frequently repeating pattern, persisting for approximately 1 s every 5 s; **F** following the emergence of sharp 2–5 phase periodic discharges, exhibiting a similar duration and cycle to the theta waves; **G** after administering a total of 150 mL of 20% lipid emulsion, showing the return of delta and alpha oscillations

The anesthetic record and EEG spectrogram throughout the course of events are shown in Fig. 1A. Due to the presence of a potentially difficult airway, awake nasotracheal intubation was performed using a local anesthetic spray (total of 10 mL of 4% lidocaine) and a continuous infusion of remifentanil at 0.075 μg/kg/min. Following successful intubation, general anesthesia and neuromuscular blockade were induced with 10 mg of remimazolam and 60 mg of rocuronium, respectively. The initial frontal EEG waveform after induction of general anesthesia exhibited delta and alpha oscillations (Fig. 1B). General anesthesia was maintained with remimazolam (30–100 mg/h), remifentanil (0.075–0.25 μg/kg/min), and intermittent boluses of fentanyl (total dose: 700 μg). Neuromuscular blockade was maintained with intermittent boluses of rocuronium, achieving a train-of-four count of less than 1 throughout the surgery.

Thirty minutes after the administration of topical airway anesthesia, continuous theta waves appeared on the frontal EEG (Fig. 1C). These theta waves were further enhanced immediately after the administration of 20 mL of 1% lidocaine with 1:100,000 epinephrine for chest wall local anesthesia (Fig. 1D). Subsequently, the theta waves transitioned into a frequently repeating pattern, persisting for approximately 1 s every 5 s (Fig. 1E). Eventually, sharp 2–5 phase periodic discharges emerged, exhibiting a similar duration and cycle to the theta waves (Fig. 1F). Following the administration of 20% lipid emulsion in divided doses of 50 mL, totaling 150 mL, the EEG normalized to delta and alpha oscillations (Fig. 1G).

During the period of EEG alterations, the bispectral index ranged from 28 to 63. The predicted effect-site concentrations of remimazolam [4], remifentanil [5], and fentanyl [6] were 660–880, 2.5–8.5, and 0–2.3 ng/mL, respectively. Although the QRS duration and corrected QT interval on the ECG were slightly prolonged, with fluctuations observed in the corrected QT interval (Fig. 1A), both normalized following lipid emulsion therapy.

The surgery was completed uneventfully in 368 min. The patient was alert and calm 8 min after the discontinuation of remimazolam infusion. He followed commands 3 min after the administration of 0.2 mg flumazenil. Following uneventful extubation, the patient remained stable and exhibited no prodromal CNS symptoms of LAST, such as dizziness or tinnitus. His postoperative course was uneventful except for right ulnar nerve palsy, likely caused by intraoperative compression of the right upper limb. He was discharged on postoperative day 10.

## Discussion

Under normal circumstances, local anesthetics block nerve conduction by inhibiting the transduction of sodium, calcium, and potassium through voltage-gated ion channels located in the cell membrane [1]. At increasing plasma concentrations, local anesthetics initially compromise cortical inhibitory pathways by disrupting inhibitory neuron depolarization [7]. This inhibition leads to excitatory clinical features, including sensory changes, muscular activation, and seizure activity. As plasma concentrations continue to rise, excitatory pathways are also affected, resulting in a depressive phase of neurological toxicity.

Given the effects of local anesthetics on multiple ion channels and the diverse CNS presentations in LAST, it is plausible that EEG monitoring could reveal a variety of abnormal signatures. EEG monitoring allows real-time assessment of cortical electrical activity, enabling clinicians to detect epileptiform discharges or other abnormal patterns. However, the effects of local anesthetics on human EEG signatures under general anesthesia are poorly documented, making it challenging to definitively attribute specific EEG alterations to LAST.

In the present case, several points suggest that the observed EEG changes may have been caused by LAST: (1) theta waves are not typically predominant in frontal EEG under the predicted effect-site concentration of remimazolam in this patient [8]; (2) lidocaine absorption through the upper airway mucosa peaks at plasma levels approximately 20–60 min after administration [9]; (3) LAST can persist for several hours following airway local anesthesia [10]; (4) theta wave power was enhanced immediately after the administration of lidocaine combined with epinephrine, which is associated with greater CNS toxicity compared to lidocaine alone [11]; (5) theta waves evolved into sharp waves, suggesting epileptiform discharges [12]; and (6) sharp waves resolved after lipid emulsion therapy, coinciding with the normalization of the QRS duration and corrected QT interval on the ECG [7]. Remifentanil and fentanyl are known to cause EEG slowing in a dose-dependent manner; however, the predicted effect-site concentrations in this patient were unlikely to have induced EEG slowing to the extent of theta wave activity [13, 14].

The diagnostic criteria for electrographic seizures, as outlined by the American Clinical Neurophysiology Society, include epileptiform discharges averaging > 2.5 Hz for ≥ 10 s (> 25 discharges in 10 s) or any pattern with definite evolution lasting ≥ 10 s [12]. In the current case, the evolution of EEG morphology was observed as follows: continuous theta waves, followed by repeating series of theta waves, and subsequently sharp 2–5 phase periodic discharges, suggesting the development of electrographic seizures.

One possible explanation for the absence of clinical convulsions is the maintained neuromuscular blockade during surgery. Despite the continuous infusion of remimazolam, a benzodiazepine recommended for managing seizures associated with LAST [1], sharp waves were observed. In response, 20% lipid emulsion was administered in divided doses under careful observation. Although it is unclear whether the neuropathophysiology observed in this patient required emergency intervention, aggressive management was undertaken due to concerns about the potential for LAST to manifest as sudden cardiovascular collapse under general anesthesia [1, 3]. Lipid emulsion therapy for LAST is associated with rare or minimal adverse effects [1, 2], and its benefits were considered to outweigh the risks. Consequently, the pronounced EEG changes and minor ECG abnormalities normalized without any complications.

In conclusion, the present case suggests that EEG monitoring following the administration of local anesthetics during general anesthesia may facilitate the early detection of LAST and the evaluation of treatment efficacy. Since perioperative frontal EEG monitoring is routinely performed to assess the depth of anesthesia, leveraging EEG for the diagnosis and management of LAST appears to be both practical and feasible. Further research is warranted to elucidate the effects of local anesthetics on EEG patterns under general anesthesia and to evaluate the clinical utility of EEG monitoring for the diagnosis and treatment of LAST in this context.

## Data Availability

The datasets used and/or analyzed during the current study are available from the corresponding author on reasonable request.

## Abbreviations

CNS: Central nervous system
ECG: Electrocardiogram
EEG: Electroencephalogram
LAST: Local anesthetic systemic toxicity
